# A Gender-based Comparison in Health Behaviors and State of Happiness among University Students

**DOI:** 10.7759/cureus.2342

**Published:** 2018-03-19

**Authors:** Rehana Rehman, Amara Zafar, Aleena Mohib, Mukhtiar Baig

**Affiliations:** 1 Department of Biological and Biomedical Sciences, Aga Khan University, Karachi.; 2 Dow Medical College, Civil Hospital Karachi, Dow University of Health Sciences, Karachi, Pakistan; 3 Faculty of Medicine, King Abdulaziz University

**Keywords:** health behaviors, smoking, dietary pattern, breast examination, dental hygiene

## Abstract

Objective

The presence of good healthy behaviors among university students is imperative for their future life. This study aimed to compare positive health behaviors and state of happiness between the two genders of Bahria University (BU), Karachi, Pakistan.

Subjects and methods

This cross-sectional study was conducted at BU, Karachi, Pakistan. A total 813 students participated in this study. The health questionnaire, adapted from Health and Behavior Survey and the Subjective Happiness Scale, was used to assess self-reported happiness. The data were analyzed using IBM SPSS version 22.

Results

Overall self-reported health behaviors were found to be more prevalent in females as compared to males, but males reported a better self-reported general health (p = 0.012). Testicular and breast self-examination was not common in both genders. Smoking was found to be more common in males (p < 0.01). Males exhibited habit of regular breakfast (p = 0.013) whereas females showed a tendency to avoid food rich in cholesterol and fat (p < 0.01) and the practice of consuming food rich in fiber was found to be more prevalent among females (p < 0.01) and they showed urge to lose weight (p < 0.01). Sleep disturbance was found in both genders, more so in females (p = 0.012). Consciousness about dental hygiene was common in females (p < 0.01). As compared to the females, more males strongly believe that they are very happy in life (p < 0.01), and they make the most out of everything in life (p < 0.01).

Conclusion

There was a diverse response to positive health behaviors and state of happiness in both genders.

## Introduction

Generally, health behavior reveals an individual's health beliefs. Specific general health behaviors are exercising consistently, consuming a balanced diet, and attaining necessary immunizations [1]. The health behaviors have potential to impact the current, as well as the future, health outcomes [2].

The prevalence of smoking and sleep disturbance is high among university students [3-5]. Wardle et al. reported in their extensive study that most females are on a diet owing to their health beliefs and weight control, resulting in making healthier food choices as compared to men [6]. The importance of early detection of breast cancer cannot be overstated. As there is the deficiency of knowledge, and its screening modalities are not readily available in our parts of the world, breast self-examination (BSE) is the only feasible way to promote early detection, preceded by extensive awareness programs [7]. On the other hand, testicular self-examination (TSE) role for early detection of testicular cancer remains controversial [8].

Health risk behaviors are behaviors, which can potentially harm a person’s health, by causing illness, injury or death. A recent study and several others have concluded that the male gender exhibits more health risk behaviors than the female gender [9], moreover, female gender is said to have higher standards of hygiene [10]. Happiness is related to emotional wellbeing that synchronizes mind with body and enables effective execution of a life free from stress. Unsurprisingly, university students are inadvertently subject to a lot of stress, and this has been depicted to influence the level of happiness of students [11]. Like other factors, one would expect to find gender-based inconsistencies in the level of happiness also, however a study stated that happiness is independent of gender [12].

Most of the health-related studies conducted in Pakistan are based on the data collected in the National Health Survey of Pakistan (1990-1994), which reported Pakistani women of all ages rated their health as poor/fair as compared to the males [13]. It is safe to say that there is a scarcity of literature on health behaviors and state of happiness based on updated and new data pertaining to the population of Pakistan. Therefore, it is important to investigate the gender-wise discrepancies regarding health behaviors and state of happiness in our local population. Hence, this study aims to compare positive health behaviors and state of happiness between the two genders of Bahria University (BU), Karachi, Pakistan.

## Materials and methods

This cross-sectional study was carried out among the students of BU in Karachi. The study was conducted from January 2012 till December 2013 after obtaining ethical approval from the Ethical Review Committee of the BU, Karachi. The sample size was calculated in open EPI software sample size calculator with 95% confidence interval and 5% margin of error. The questionnaire was distributed to multiple institutes of BU including Medical and Dental College, Engineering, Business Management, Social sciences and Environmental sciences institutes. Data were collected by a self-administered anonymous questionnaire in a classroom situation after obtaining informed consent. Students were further given information that the concerned survey responses were related to health and wellbeing. Responses were acquired by convenient sampling technique, and 813 complete responses were included. The health questionnaire, adapted from Health and Behavior Survey [14], was used for data collection. The demographic information like age, gender, year of study, residence, marital status, and smoking history was obtained. The Subjective Happiness Scale was used to assess self-reported happiness, which uses four questions to assess an individual’s overall happiness as measured through self-evaluation [15].

Statistical analysis

The data were analyzed using IBM Statistical Package for the Social Sciences (IBM SPSS Statistics for Windows, Version 20.0, Armonk, New York). Pearson Chi-Square test was applied to find associations between the health behaviors and gender differences. The p-value < 0.05 was considered significant in all cases. Tables were constructed using Microsoft Excel 2016.

## Results

The sample included 813 students (41.1% males and 58.9% females) with a mean age of 19.9 years (SD = 1.8). Almost half of the participants (53.1%) were from first years, while 26.2%, 7.6%, 12.7%, and 0.4% were from second, third, fourth and fifth years, respectively. A large majority (73.7%) were medical students, 7.5% engineering and 18.8% in other courses. A total of 42.3% of the women self-reported that they knew how to examine their breasts for lumps while more than half of them did not, and 60.7% of the women had never examined their breast while a quarter of them examine their breasts at least once or twice each year and 14.4% women examined their breasts more than twice a year. Only 28.5% of men knew how to examine their testicle for a lump (results not shown in table).

Table 1 describes that overall males showed better self-reported general health (p = 0.01), but more of them indulged in smoking as compared to women (p < 0.01). The habit of eating breakfast was found to be more common in males (p = 0.01), but other healthy behaviors like conscious avoiding of cholesterol and food rich in fat and consuming food rich in fiber was found to be more prevalent among females (p < 0.01 and p < 0.01, respectively). Twice as many women as men were found to be trying to lose weight; hence, body dissatisfaction was common in females and was present to a lesser extent in the males also (p < 0.01). Sleep disturbance was found in both genders but more prevalent in females (p = 0.01). Males reported wearing seatbelts more than females but the difference between the two was not found to be significant (p = 0.36). Females were found to be more conscious of dental hygiene (p < 0.01).

**Table 1:** Gender-wise comparison of self-reported health behaviors by using Health and Behavior Survey Questionnaire. *p < 0.05 was considered significant using Pearson Chi Square test.

Characteristics		Male	Female	p-value
		n (%)	n (%)	
Self-reported general health	Excellent	59 (17.4)	60 (12.7)	0.012*
	Very good	125 (36.8)	153 (32.3)	
	Good	119 (35.0)	186 (39.3)	
	Fair	30 (8.8)	70 (14.8)	
	Poor	7 (2.1)	4 (0.9)	
Tobacco products use currently	Yes	72 (21.2)	36 (7.6)	<0.01*
	No	268 (78.8)	437 (92.4)	
How often use of tobacco products in the past month?	Once or twice a week	24 (33.3)	24 (66.7)	0.013*
	Weekly	19 (26.4)	5 (13.9)	
	Almost daily	8 (11.1)	2 (5.6)	
	Daily	21 (29.2)	5 (13.9)	
How often do you eat breakfast?	Almost everyday	214 (62.9)	260 (55.0)	0.013*
	Sometimes	86 (25.3)	123 (26.0)	
	Rarely or never	40 (11.8)	90 (19.0)	
Do you make a conscious effort to avoid eating foods that contain fat and cholesterol?	Yes	111 (32.6)	221 (46.7)	<0.01*
	No	229 (67.4)	252 (53.3)	
Do you make a conscious effort to eat foods that are high in fiber?	Yes	117 (34.4)	218 (46.1)	<0.01*
	No	223 (65.6)	255 (53.9)	
Are you trying to lose weight?	Yes	84 (24.7)	225 (47.6)	<0.01*
	No	256 (75.3)	248 (52.4)	
Are you dieting to lose weight?	Yes	27 (7.9)	81 (17.1)	<0.01*
	No	313 (92.1)	392 (82.9)	
Self reported weight	Very overweight	11 (3.2)	27 (5.7)	<0.01*
	Slightly overweight	75 (22.1)	189 (40.0)	
	About right	199 (58.5)	196 (41.4)	
	Slightly underweight	45 (13.2)	49 (10.4)	
	Very underweight	10 (2.9)	12 (2.5)	
Sleep disturbances	None	149 (43.8)	162 (34.2)	0.012*
	Mild	92 (27.1)	139 (29.4)	
	Moderate	63 (18.5)	130 (27.5)	
	Severe	31 (9.1)	34 (7.2)	
	Extremely/cannot do	5 (1.5)	8 (1.7)	
Do you wear seatbelt?	All the time	52 (15.3)	60 (12.7)	0.366
	Some of the time	122 (35.9)	153 (32.3)	
	Never	136 (40.0)	215 (45.5)	
	I don’t ride in cars	30 (8.8)	45 (9.5)	
Tooth brush	Twice or more a day	129 (37.9)	277 (58.6)	<0.01*
	About once a day	193 (56.8)	184 (38.9)	
	Less than once a day	8 (2.4)	9 (1.9)	
	Seldom or never	10 (2.9)	3 (0.6)	

Table 2 describes that more than half of the males and females considered themselves as happy with a 4% male predominance. Of the remaining, it was seen that more males as compared to females said that they did not consider themselves as happy and twice as many females as men had no opinion on this (p < 0.01). More men as compared to women considered themselves as happy when they compared themselves to their peers (p < 0.01). More males than females reported extreme agreement or disagreement to the statement that they enjoy life regardless of what is going on (p < 0.01). When asked if it is true that they might not seem as happy as they really might be, it was noticed that half of the females in the sample disagreed to it while a quarter agreed to it and lesser males as compared to females disagreed with the statement (p = 0.01).

**Table 2:** Gender-wise comparison of self-reported happiness by using subjective happiness scale. *p < 0.05 was considered significant using Pearson Chi Square test.

Characteristics		Male	Female	p-value
		n (%)	n (%)	
In general, I consider myself a very happy person	Strongly disagree	48 (14.1)	26 (5.5)	<0.01*
	Disagree	22 (6.5)	33 (7.0)	
	Neither disagree or agree	49 (14.4)	124 (26.2)	
	Agree	130 (38.2)	220 (46.5)	
	Strongly agree	91 (26.8)	70 (14.8)	
Compared to most of my peers, I consider myself more happy	Strongly disagree	34 (10.0)	32 (6.8)	<0.01*
	Disagree	33 (9.7)	33 (7.0)	
	Neither disagree nor agree	68 (20.0)	146 (30.9)	
	Agree	121 (35.6)	190 (40.2)	
	Strongly agree	84 (24.7)	72 (15.2)	
Some people are generally very happy. They enjoy life regardless of what is going on, getting the most out of everything. To what extent does this characterization describe you?	Strongly disagree	42 (12.4)	38 (8.0)	<0.01*
	Disagree	39 (11.5)	61 (12.9)	
	Neither disagree nor agree	87 (25.6)	130 (27.5)	
	Agree	89 (26.2)	186 (39.3)	
	Strongly agree	83 (24.4)	58 (12.3)	
Some people are generally not very happy. Although they are not depressed, the never seem as happy as they might be. To what extent does this characterization describe you?	Strongly disagree	58 (17.1)	114 (24.1)	0.013*
	Disagree	90 (26.5)	135 (28.5)	
	Neither disagree nor agree	94 (27.6)	102 (21.6)	
	Agree	71 (20.9)	102 (21.6)	
	Strongly agree	27 (7.9)	20 (4.2)	

## Discussion

The present survey compared the general health status of male and female students by asking for a self-rating. It has been shown that self-rating of health can be considered reliable, valid and has high predictive power for a variety of illnesses and conditions [16].

Two-thirds of the women in our study reported that they are aware of how to self-examine their breast for a lump. A study in Rawalpindi in 2009 reported a total of 28.3% of the females in the sample being aware of BSE [7]. A quarter of the female subjects in the study practiced BSE at least once or twice each year with about 14.4% of females practicing it more than twice a year. In a study in Iran in 2008, they reported that just one-third of the subjects had executed BSE in the past and 7.1% of them did it on monthly basis [17].

In our study, only 28.5% of men knew how to examine their testicle for a lump. In a study in Turkey, only a minor percentage of the students pointed out that they obtained information on TSE and 17.7% have executed the training of TSE previously [18].

The results of our study indicated that more males as compared to females perceived their health status as excellent and very good. Similarly, a study in Italy among the university students reported lower self-perceived health by the women [19]. However, a study conducted among the school students reported poor self-perceived health status among boys [20].

Males in our study outweighed the females in the use of tobacco by a ratio of 2:1; an almost similar ratio, 1.92:1, was seen in a study in a private medical college in Belgaum [4]. However, in King Saud University a much greater difference was seen between the two genders, with males using tobacco five times more as compared to the females [21]. Although tobacco use has been found to be more in males than females in most of the studies, but generally, a rapidly increasing rate is seen among young women especially in developing countries as explained by Khor et al., in their study [22]. More females were found to skip breakfast as compared to the males with more males doing breakfast almost every day. Similar results were obtained among school adolescents in Iran [23].

Approximately half of the female population in our study made conscious efforts to eat food that is low in cholesterol and rich in fiber while only one-third of the males observed this behavior. Studies reporting results from 23 countries quoted similar findings and stated that the women gave great importance to healthy eating and are likely to be dieting [6]. In line with the above stated selective eating found among the females in our study, it was also seen that about half the females are trying to lose weight and approximately two-fifths of those wanting to lose weight said they are on diet control for it. Fast food and soft drinks consumption is linked to weight gain [24] and it was observed in a study among adolescents in Syria that males were found to consume larger proportion of fast food and drinks as compared to the girls of the same age group and the difference being almost twice between the gender [25] further showing healthier food choices made by women as compared to men.

A greater body weight dissatisfaction was seen in females with more than half of the females in the sample being discontent with their weight while approximately 60% of the males were satisfied with their body weight. Similar body weight dissatisfaction was seen in Pharmacy students in Poland where females overestimated their body weight while males underestimated their body weight [26].

Sleep disturbance was more common among females. A study on sleep patterns among the college students in Taiwan reported poorer sleep quality among the female students [5]. Another study among US college students stated that most of the students faced some form of sleep disturbance with females being more commonly affected [27]. Female gender was found to have higher standards of dental hygiene with more than half of the female in the study brushing their teeth at least twice a day. Similar results were seen in a study among dental students in Palestine where females were seen to observe positive dental health behaviors more than their male counterpart [28].

The Subjective Happiness Scale was used to evaluate self-reported happiness. The scale uses four questions to evaluate a person's overall happiness as measured via self-evaluation [15]. We observed significant gender differences in each question of the scale. It was seen that more males when compared to females strongly believed that they are generally very happy in life, when compared to their peers they are happier than them and that they make the most out of everything in life regardless of what is going on. On the contrary, a recent study in Iran concluded that the state of happiness is independent of gender [12].

There is a limited data available regarding the health behaviors and state of happiness observed in Pakistan, especially among the university students. Our study will serve as a basis for health behavior intervention, to improve the health of the students and the general population of Pakistan.

Limitations of study

Our sample included students from a single university, which cannot be representative of universities all over Pakistan. Replication of the study with a well-distributed sample can provide a better comparison of eating habits, health behaviors and state of happiness and satisfaction between male and female students all over Pakistan.

## Conclusions

There was a diverse response to positive health behaviors and state of happiness in both genders. Males exhibited habit of regular breakfast whereas females showed a tendency to avoid food rich in fiber and fat and had an urge to lose weight. Sleep disturbance was found in both genders more so in females. Consciousness about dental hygiene was common in females. The element of happiness was prevalent in males. We suggest longitudinal studies to confirm our results.
